# A national evaluation of the management practices of hemorrhoidal disease in the Netherlands

**DOI:** 10.1007/s00384-018-3019-5

**Published:** 2018-03-15

**Authors:** Robin R. van Tol, Marieke P. A. Bruijnen, Jarno Melenhorst, Sander M. J. van Kuijk, Laurents P. S. Stassen, Stéphanie O. Breukink

**Affiliations:** 1 Department of Surgery and Colorectal Surgery, Academic Hospital Maastricht, Maastricht University Medical Center, PO box 5800, 6202 AZ Maastricht, The Netherlands; 2 Department of Clinical Epidemiology and Medical Technology Assessment, Maastricht University Medical Centre, Maastricht, the Netherlands

**Keywords:** Hemorrhoids, Survey, Therapy, Surgical, Treatment algorithm

## Abstract

**Purpose:**

In this study, we describe current practices in the management of hemorrhoidal disease in the Netherlands.

**Methods:**

A validated online survey was performed among Dutch surgeons and residents treating hemorrhoidal disease. Contact details were retrieved from the Dutch Association for Surgery resulting in 619 contacts. Only doctors who were treating hemorrhoidal disease regularly were asked to complete the questionnaire. The following items were assessed: initial treatment, recurrence, complications, and follow-up.

**Results:**

In total, 133 respondents completed the survey. Ninety percent of the respondents started with rubber band ligation (RBL) as the first treatment in low-grade hemorrhoidal disease. In case of recurrence, 64% of the respondents repeated RBL three times before switching to a more invasive treatment modality. In grade III hemorrhoidal disease, the respondents preferred more invasive techniques: a sutured hemorrhoidopexy was performed in 24%, Doppler-guided hemorrhoidal artery ligation (DG-HAL) in 9%, stapled hemorrhoidopexy in 19%, and the traditional hemorrhoidectomy in 31% of the patients, respectively. The majority of the respondents (39%) reported a mild complication in 5–10% of the patients. The most reported complication was pain. Nearly all the respondents (98%) reported a major complication in less than 5% of the patients. The majority of the patients (57%) were seen in outpatient clinics 6 weeks post-treatment.

**Conclusion:**

This Dutch survey showed areas of common practice for primary treatment of hemorrhoidal disease. However, it also demonstrated varying practices regarding recurrent hemorrhoidal disease. Practical guidelines are required to support colorectal surgeons in the Netherlands.

## Introduction

Hemorrhoidal disease is a common pathology with prevalence rates of up to 44% within the general population [1–3]. Hemorrhoids are usually classified by their location and by the presence and severity of prolapse. The most widely accepted classification is the Goligher classification [4]. Initial treatment of grades I–II hemorrhoidal disease is quite uniform. Conservative treatment including diet, lifestyle changes, and application of topical ointments is mostly offered as a first step [5–7]. In case of persistent symptoms, patients are usually treated with rubber band ligation (RBL) [8]. However, it is still unclear what the best next treatment modality is in case of recurrence after several failed RBL attempts.

Grades III and IV hemorrhoidal disease is often treated in a more invasive way, thereby skipping the first two steps. Similar to recurrence after RBL for grade I and II disease, there remains a debate what the best (surgical) treatment option is in case of recurrence.

Over the past two decades, knowledge of the anatomy of hemorrhoids has improved, leading to the introduction of new surgical technologies. This was accompanied by many studies comparing several surgical treatments including the Doppler-guided hemorrhoidal artery ligation (DG-HAL) with or without recto-anal repair (RAR) [9–11], a sutured hemorrhoidopexy [12, 13], the stapled hemorrhoidopexy (SH) [14–16], and the traditional hemorrhoidectomy [17–20].

However, systematic reviews [21, 22] and guidelines [23, 24] **including a Dutch guideline of Dunker et al. (published in a Dutch guideline database)** highlighted the lack of a high level of evidence which is mandatory to develop an optimal treatment algorithm. Recently, two high-quality RCTs have been published [25, 26]. Results of these studies may not have been implemented in clinical practice yet.

Besides, as many studies use different outcomes assessing treatment effect, data of these studies cannot easily be compared or pooled into a single inference. As a result, it is difficult to determine what treatment yields the highest clinical benefit for each grade or what treatment is advocated in case of recurrence.

The aim of the present study was to assess current practice in (surgical) treatment of hemorrhoidal disease using a national survey among officially registered Dutch colorectal consultants, fellows, and residents in the Netherlands. Besides the complications for each treatment and outcome, parameters to determine treatment success were recorded.

## Materials and methods

### Design of the survey

Two surgical residents (RS, RT) formulated the questions of the survey. These questions were edited by a colorectal surgeon (SB). After making adjustments, the survey was reviewed by a second colorectal surgeon (LS).

The survey was created using a validated web-based program [27]. The survey consisted of 30 items: 13 multiple- choice questions, 14 optional questions, and three open-ended questions with a total word count of 1144 words. The questionnaire was developed in Dutch [see Appendix 1 for a version translated to English]. In order to check comprehensibility and content validity of the survey, several rounds of pilot testing were conducted before its actual distribution.

#### Survey distribution

We distributed the survey among officially registered Dutch colorectal consultants, fellows and residents. Contact details were retrieved from the Dutch Association for Surgery resulting in 619 contacts. Only doctors who were treating hemorrhoidal disease regularly at the time of the questionnaire, irrespective of the number of years of experience with the treatment, were asked to complete the questionnaire. A personalized e-mail with a link to the web-based survey was sent to each of them, and a reminder was sent 1 week later.

### Data analysis

Only completed surveys were included in the analysis. Characteristics of the doctors, treatments used for primary and recurrent disease stratified by grade, and the outcome parameters were all described using absolute value and percentage. The complications were estimated as cumulative incidence.

All analyses were performed in IBM SPSS version 22.0.

### Definitions

Conservative treatment consisted of diet, lifestyle changes, and application of topical ointments. Minimally invasive treatment consisted of laser therapy, Doppler-guided hemorrhoidal artery ligation (DG-HAL) (with recto-anal repair (RAR)) or a sutured hemorrhoidopexy. **The term “sutured hemorrhoidopexy” we used in this manuscript is similar to RAR or suture mucopexy described in the literature. However, the term sutured hemorrhoidopexy reflects more precisely the surgical technique; a suture is used for lifting the hemorrhoidal complex to its origin.**

Invasive treatment consisted of the stapled hemorrhoidopexy and traditional hemorrhoidectomy.

We asked two questions regarding complications. First, we asked the respondents “how often did you see a mild or severe complication after treatment for hemorrhoidal disease?” The respondents could choose between 1 and 5% or 5–10% or 10–20% or more than 20%. The second question was: “did you experience ‘no’ or ‘mild’ or ‘severe’ complications after use of either RBL, minimally invasive treatment, or invasive treatment?” This question allowed respondents to select more than one answer.

## Results

### Characteristics respondents

Background features of the respondents are shown in Table 1. A total of 100 participants returned a completed survey. The majority of hemorrhoidal disease was treated by the department of surgery (82.4%). Patients were mostly seen in outpatient clinics for the first time by a resident (44.2%) or consultant (37%).

**Table 1:** Background features of respondents

Background features	*N*	Percent
Characteristics of respondents		
Gender		
Male Female	76 24	76 24
Function participants		
Consultant	84	84
Fellow Resident	5 11	5 11
Years of experience hemorrhoid treatment		
1–5 years 5–10 years 10–20 years > 20 years	11 26 39 24	11 26 39 24
Type of hospital		
District University Private clinic	85 12 3	85 12 3
Treatment by the department		
Surgery Gastroenterology Dermatology	82 9 9	82 9 9
Contact first visit outpatient clinic		
Consultant Fellow Resident Nurse practitioner	38 12 44 6	38 12 44 6

## Treatment for primary disease

### Primary treatment of grade I disease

**Fig. 1:**
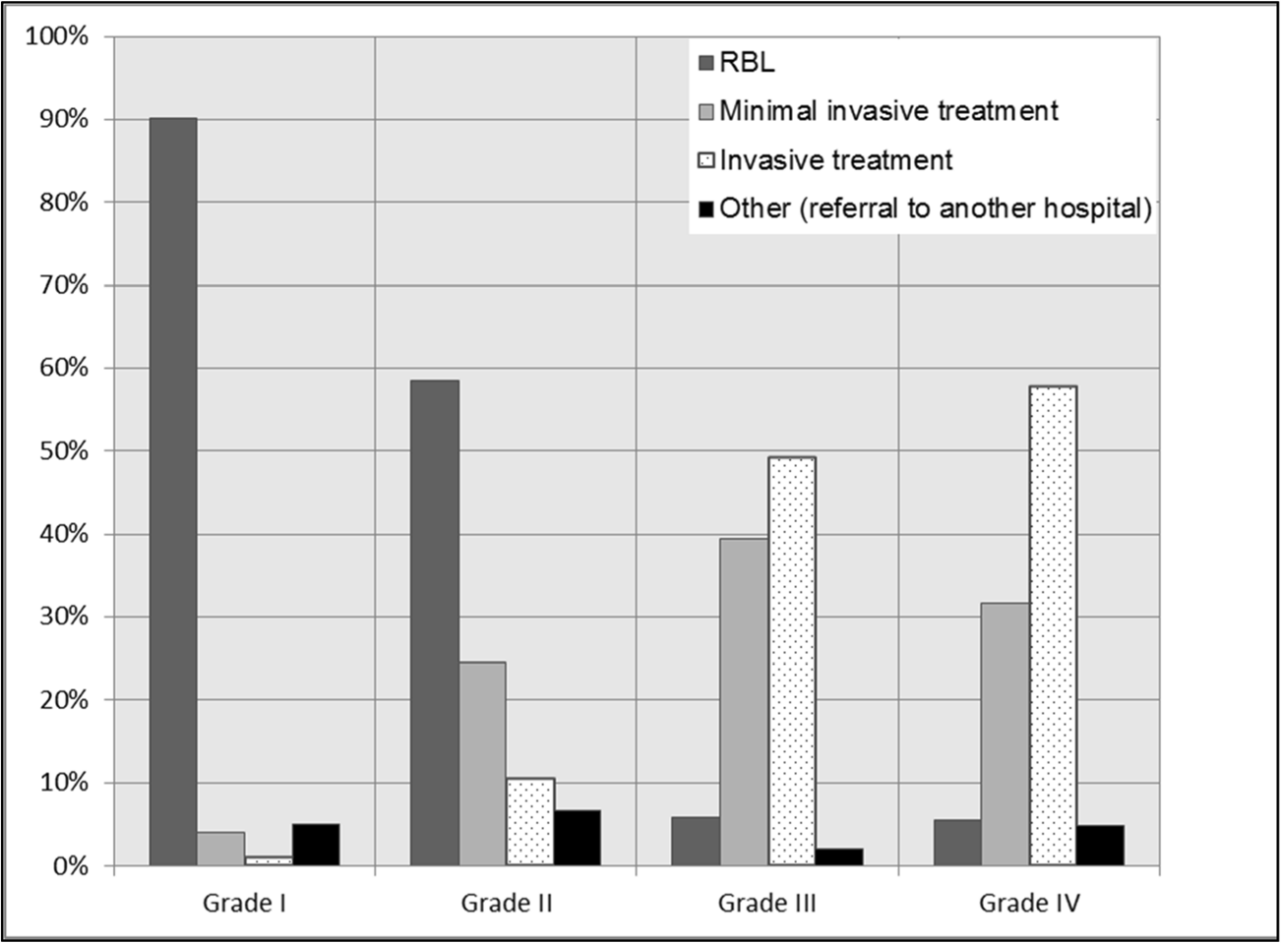
The use of rubber band ligation (RBL), minimal invasive treatment (laser therapy or sutured hemorrhoidopexy or Doppler-guided hemorrhoidal artery ligation (DG-HAL)), or invasive treatment (stapled hemorrhoidopexy or traditional hemorrhoidectomy) for grades I–IV hemorrhoidal disease according to the Goligher classification

**Fig. 2:**
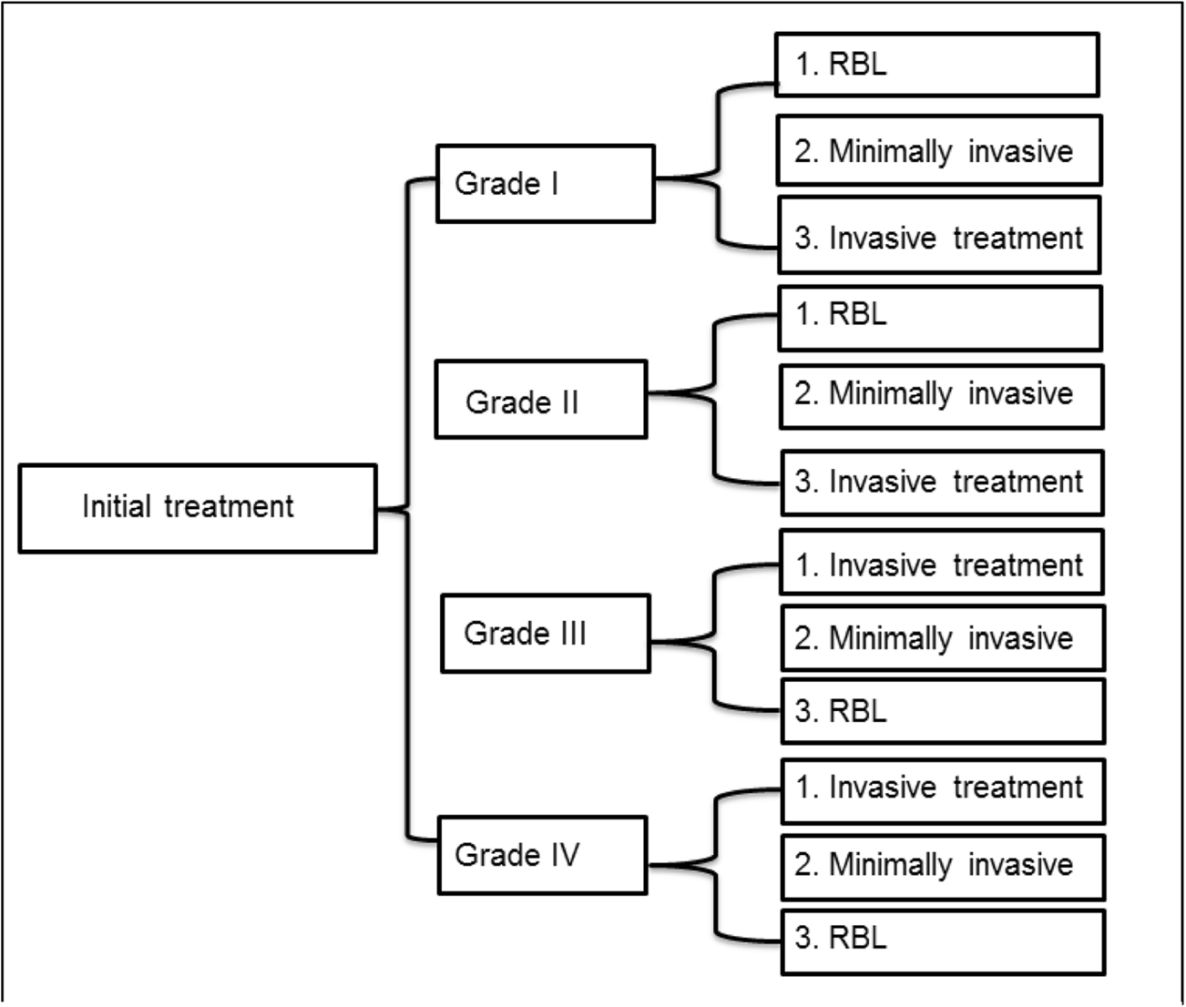
Flow diagram: initial treatment for grades I–IV hemorrhoids (RBL, rubber band ligation; minimal invasive treatment, laser therapy or sutured hemorrhoidopexy or DG-HAL; and invasive treatment, stapled hemorrhoidopexy and traditional hemorrhoidectomy)

Respondents used RBL in 90% of the patients as the first treatment modality. Regarding minimally invasive treatment, respondents used laser therapy in < 1% and the sutured hemorrhoidopexy in 3% of the patients. Regarding invasive treatment, respondents used the stapled hemorrhoidopexy in 1% of the patients (Figs. 1 and 2).

### Primary treatment of grade II disease

Fifty-nine percent of the respondents treated their patients with RBL. Regarding minimally invasive treatment, respondents used laser therapy in 4%, the sutured hemorrhoidopexy in 10%, and the DG-HAL in 10% of the patients. Regarding invasive treatment, respondents used the stapled hemorrhoidopexy in 2% and the traditional hemorrhoidectomy in 9% of the patients (Figs. 1 and 2).

### Primary treatment of grade III disease

Six percent of the respondents used RBL for grade III hemorrhoidal disease. Regarding minimally invasive treatment, respondents used laser therapy in 4%, sutured hemorrhoidopexy in 24%, and the DG-HAL in 15% of the patients. Regarding invasive treatment, respondents used the stapled hemorrhoidopexy in 19% and traditional hemorrhoidectomy in 31% of the patients (Figs. 1 and 2).

### Primary treatment of grade IV disease

Ninety percent of the respondents performed a (minimally) invasive treatment consisting of laser therapy in 2%, sutured hemorrhoidopexy in 21%, DG-HAL in 10%, stapled hemorrhoidopexy in 21%, and traditional hemorrhoidectomy in 37% of the patients (Figs. 1 and 2).

## Treatment for recurrence after initial therapy

### Recurrence after primary treatment of grade I disease

**Fig. 3:**
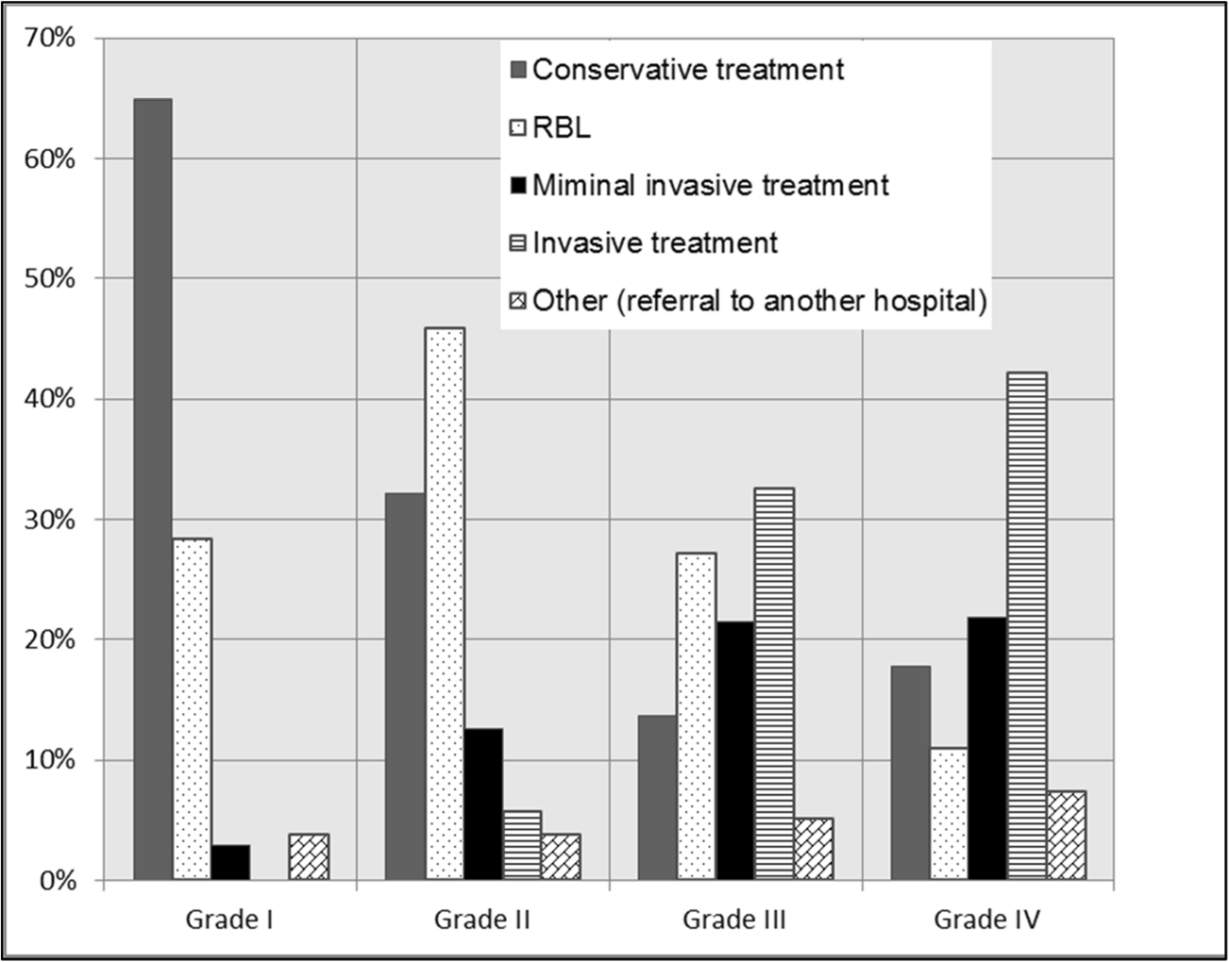
The use of conservative treatment, rubber band ligation (RBL), minimal invasive treatment (laser therapy or sutured hemorrhoidopexy or DG-HAL), or invasive treatment (stapled hemorrhoidopexy or traditional hemorrhoidectomy) in recurrent grades I–IV hemorrhoidal disease according to the Goligher classification

**Fig. 4:**
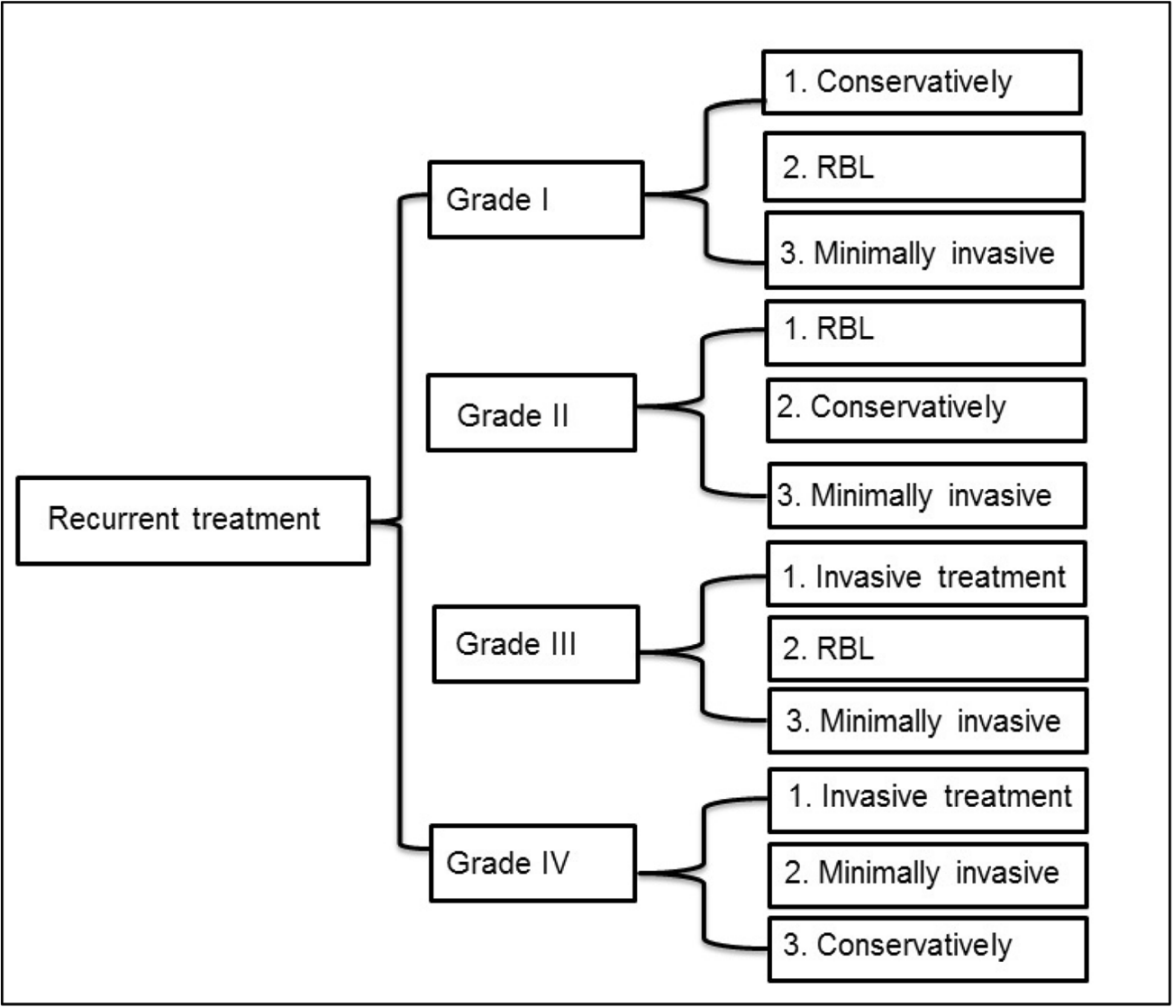
Flow diagram: treatment in case of recurrent hemorrhoids for grades I–IV hemorrhoids (RBL, rubber band ligation; minimally invasive treatment: laser therapy, sutured hemorrhoidopexy, or DG-HAL; and invasive treatment, stapled hemorrhoidopexy and traditional hemorrhoidectomy)

If complaints persisted in grade I hemorrhoidal disease, 65% of the respondents used conservative treatment. Twenty-eight percent of the respondents performed RBL. Regarding minimally invasive treatment, laser therapy is used < 1% and the sutured hemorrhoidopexy in 2% of the patients (Figs. 3 and 4).

### Recurrence after primary treatment of grade II disease

Respondents chose for conservative treatment in 32% and for RBL in 46% of the patients. Regarding minimally invasive treatment, laser therapy was used in 1%, the sutured hemorrhoidopexy in 8%, and DG-HAL in 4% of the patients. Regarding invasive treatment, respondents used the stapled hemorrhoidopexy in 2% and the traditional hemorrhoidectomy in 4% of the patients (Figs. 3 and 4).

### Recurrence after primary treatment of grade III disease

Respondents used conservative treatment in 14% and RBL in 27% of the patients. Regarding minimally invasive treatment, respondents used laser therapy in 2%, the sutured hemorrhoidopexy in 13%, and DG-HAL in 6% of the patients. Regarding invasive treatment, respondents used the stapled hemorrhoidopexy in 11% and traditional hemorrhoidectomy in 21% of the patients (Figs. 3 and 4).

### Recurrence after primary treatment of grade IV disease

Recurrence in grade IV hemorrhoidal disease was treated conservatively by the respondents in 18% and RBL in 11% of the patients. Regarding minimally invasive treatment, respondents used laser therapy in 2%, the sutured hemorrhoidopexy in 14%, and DG-HAL in 5% of the patients. Regarding invasive treatment, respondents used the stapled hemorrhoidopexy in 14% and traditional hemorrhoidectomy in 28% of the patients (Figs. 3 and 4).

In case RBL failed, 80% of the respondents reported that a patient underwent a new RBL after a mean of 6.4 weeks. Sixty-four percent of the respondents performed two to three attempts of RBL before switching to another treatment option. In 41% of the cases, a RBL was performed by residents.

## Complications

**Fig. 5:**
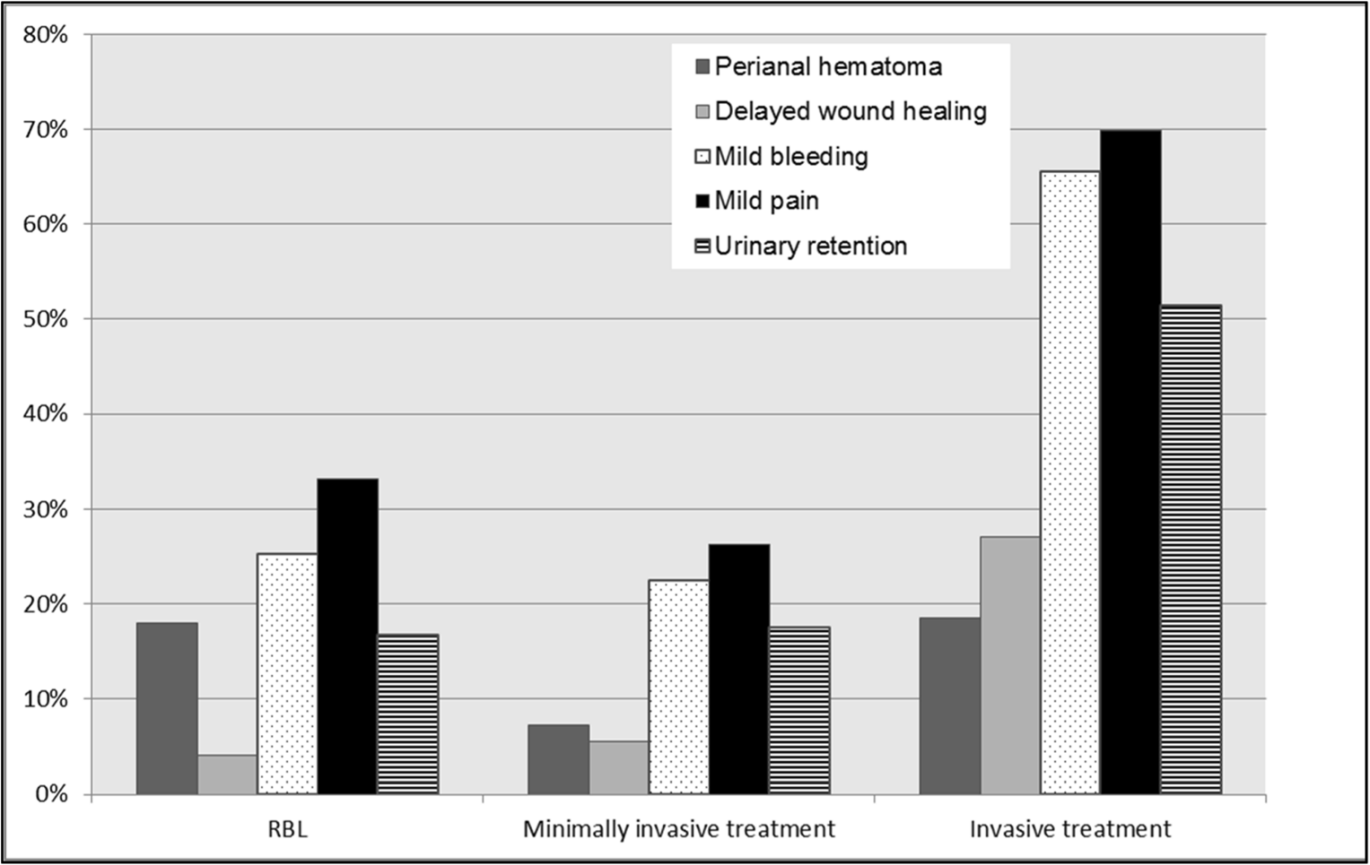
Cumulative reported mild complications (Clavien-Dindo 1–2) after RBL (rubber band ligation), minimal invasive treatment (laser therapy or sutured hemorrhoidopexy or DG-HAL), and invasive treatment (stapled hemorrhoidopexy and traditional hemorrhoidectomy)

The majority of respondents (39%) reported a mild complication in 5–10% of the patients after any treatment for hemorrhoidal disease. In Fig. 5, the cumulative reported mild complications are shown for RBL, minimally invasive treatment, and invasive treatment.

**Fig. 6:**
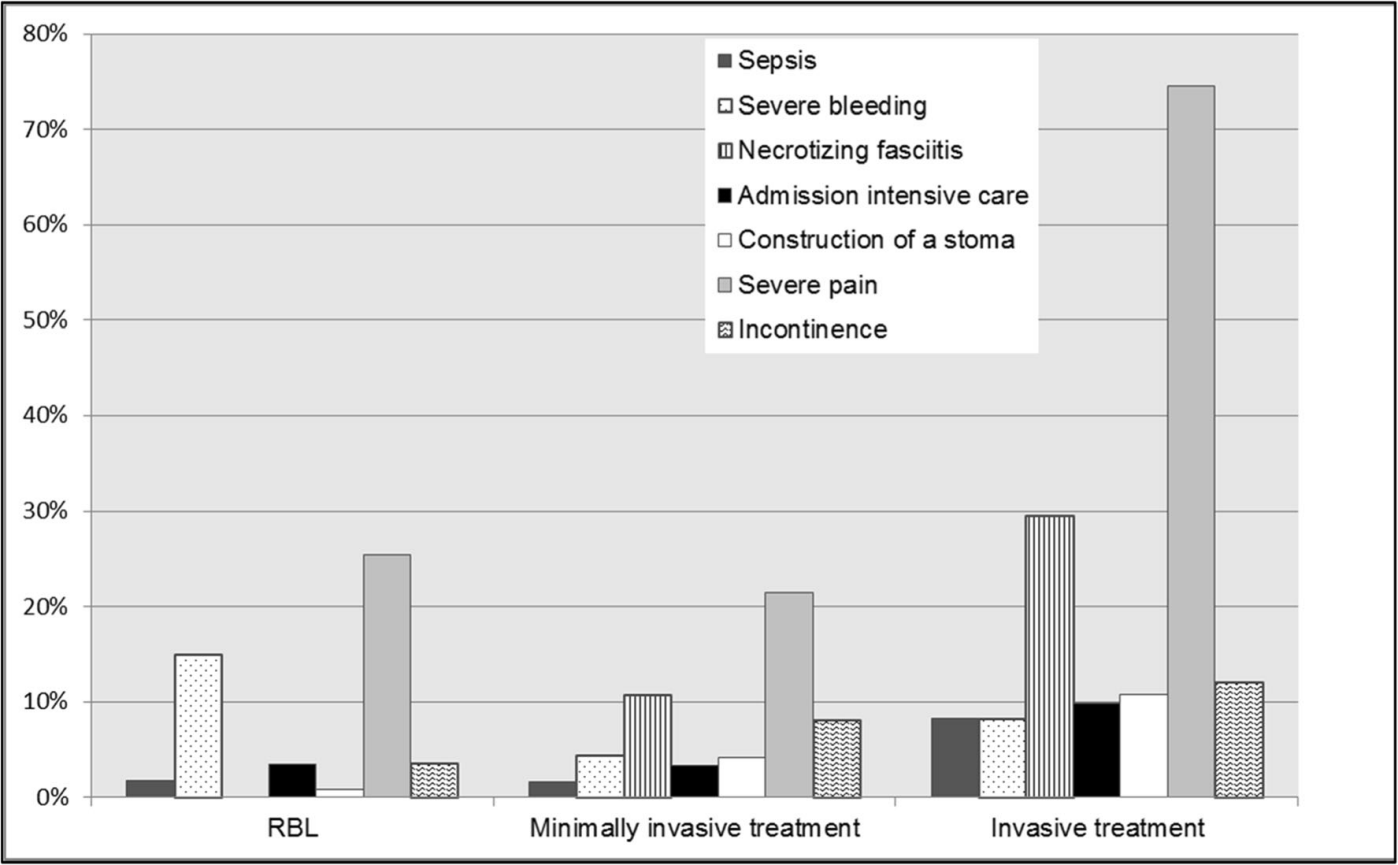
Cumulative reported severe complications (Clavien-Dindo 3–5) after RBL (rubber band ligation), minimal invasive treatment (laser therapy or sutured hemorrhoidopexy or DG-HAL) and invasive treatment (stapled hemorrhoidopexy or traditional hemorrhoidectomy)

Ninety-eight of the respondents reported major complications. Major complications occurred in less than 5% of the patients after any treatment for hemorrhoidal disease. In Fig. 6 the cumulative reported severe complications are shown for RBL, minimally invasive treatment, and invasive treatment.

## Follow-up

**Fig. 7:**
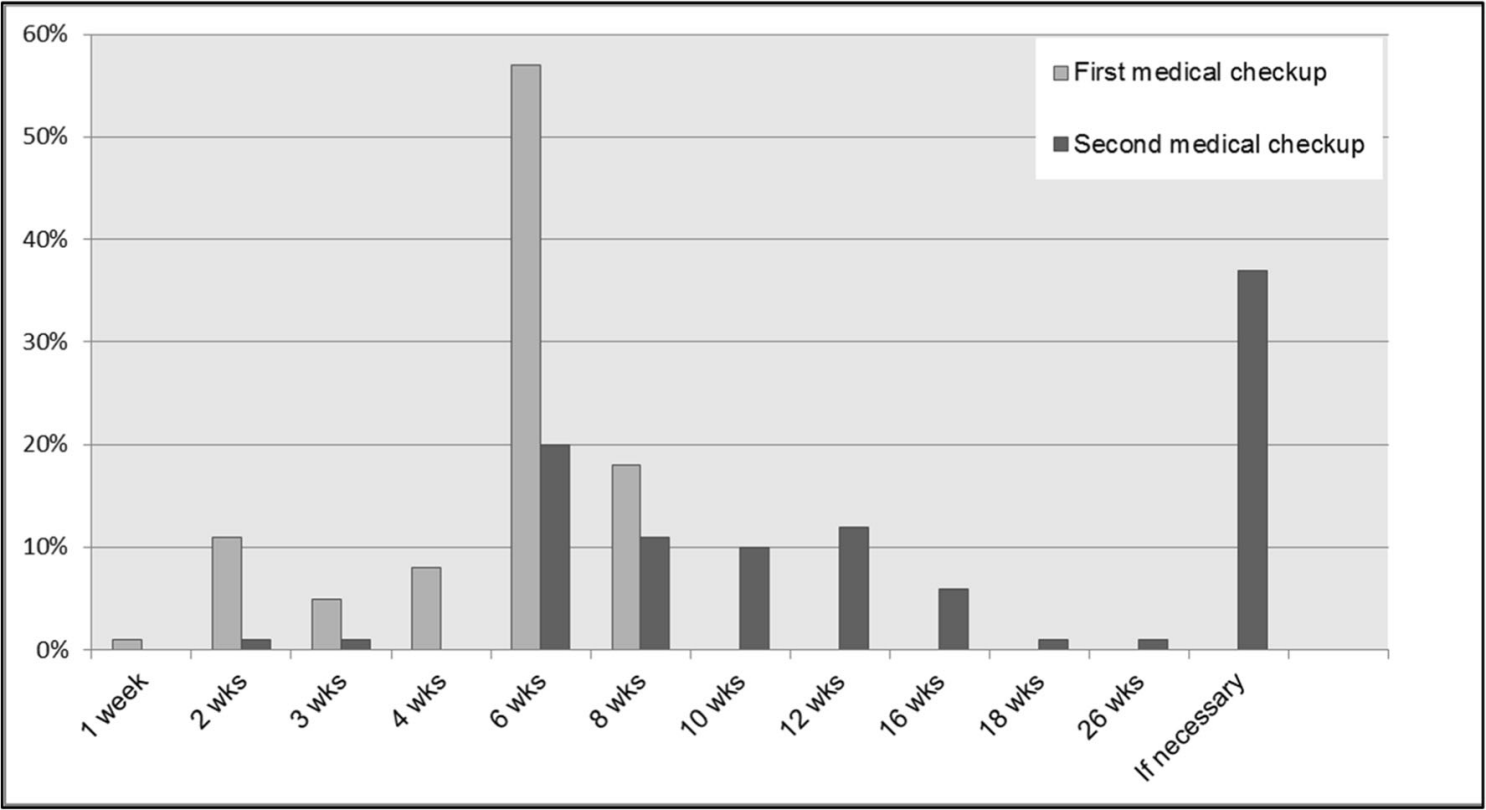
First and second outpatient visits after initial treatment for grades I–IV hemorrhoidal disease

Follow-up schedules were quite uniform. Eighty-five percent of the patients were seen 6 weeks after treatment [Fig. 7]. As the primary outcome of success, several definitions were used: 42.5% of the respondents used “patient satisfaction” and 36.7% “absence of complaints”.

## Discussion

This Dutch survey showed areas of common practice for primary treatment of hemorrhoidal disease. However, it also demonstrated more varying practices regarding recurrent hemorrhoidal disease.

Grades I and II hemorrhoidal disease were mostly treated conservatively or with RBL according to the respondents. This is in accordance with several studies and guidelines describing the optimal treatment for low-grade hemorrhoidal disease [28–30]. A review, describing seven RCTs with a total number of 378 patients, confirmed better outcome in patients with grades I–II hemorrhoidal disease who used increased fiber intake [31]. In a recent RCT, comparing RBL with hemorrhoidal artery ligation (HAL) in 370 patients with grades II and III hemorrhoidal disease, they showed that HAL resulted in fewer recurrences. However, recurrence was similar to repeat RBL [32].

In grades III and IV hemorrhoidal disease, most respondents preferred (minimally) invasive treatment instead of RBL as the first treatment option. Recently, two RCTs compared the minimally invasive treatment options Doppler-guided hemorrhoidal artery ligation (DG-HAL) combined with a suture mucopexy versus a suture mucopexy alone. They showed low recurrence rates of 2–10% after 12–24 months follow-up for the suture mucopexy group [12, 13, 33]. However, long-term results of the suture mucopexy are limited: only one RCT fulfilled a follow-up of 2 years [13]. Despite the good results, the recurrence rate was high for grade IV hemorrhoidal disease (11–59%) [22, 34].

Respondents used the stapled hemorrhoidopexy or traditional hemorrhoidectomy more often in patients with grades III and IV hemorrhoidal disease than in patients with grades II and III hemorrhoidal disease. A meta-analysis of almost 1000 patients demonstrated a higher long-term recurrence rate of 42% in patients undergoing a stapled hemorrhoidopexy compared to the traditional hemorrhoidectomy (25%) [26, 35]. Recently, Watson et al. conducted a multicenter RCT, comparing stapled hemorrhoidopexy to traditional excisional surgery in 777 patients with grade III hemorrhoidal disease. The overall quality of life was significantly better after traditional hemorrhoidectomy with a follow-up of 24 months. They concluded that the traditional hemorrhoidectomy is superior for the primary management of grades II–IV hemorrhoidal disease compared to the stapled hemorrhoidopexy [26].

In case of recurrent grades I and II hemorrhoidal disease, most respondents used conservative treatment or performed RBL. For recurrent grades III and IV hemorrhoidal disease, respondents used more often invasive treatments. As there are to our knowledge no studies focusing on what to do in case of recurrent hemorrhoidal disease, there remains a debate what the next step should be in these patients.

Ideally, an international guideline will be conducted for primary and recurrent treatment of hemorrhoidal disease. A barrier for such treatment algorithm is the lack of a known uniformly accepted core outcome set (COS). Comparing data and pooling results of clinical trials used in evidence-based health care, can only be conducted if outcomes are comparable. Therefore, we started the development of a COS assessing the treatment for hemorrhoidal disease in clinical trials [36]**.**

Some limitations of this study should be noted. Firstly, the grading of hemorrhoids can be surgeon dependent. This may result in a wide variation in classification of the severity of hemorrhoids. Secondly, the frequency of grade IV hemorrhoids is less common than the grades I, II, and III hemorrhoids. Some respondents may not have clinical experience in the treatment of grade IV hemorrhoids. Thirdly, due to the lack of a central database comprising detailed information of Dutch surgeons, the survey was sent to all Dutch colorectal consultants, fellows, and surgical residents of the Dutch Association for Surgery. They were requested to reply only in case the respondent was treated for hemorrhoidal disease regularly. This approach lacks insight in the exact response of the target population. Fourthly, due to the nature of surveys: respondents represent an intrinsic selection bias and answer options may be interpreted differently by different responders. Fifthly, the study surveyed only Dutch colorectal consultants and residents; therefore, the results cannot be generalized to surgeons worldwide. In most countries, daily care for patients with hemorrhoidal disease is conducted by surgeons. Therefore, in our survey we only assessed the surgical point of view regarding hemorrhoidal disease treatment. But we are aware that in some countries other healthcare professionals like gastroenterologists and dermatologists are involved in daily care for these patients.

## Conclusion

This survey showed that there remains considerable variation in the treatment of hemorrhoidal disease, especially in case of recurrence, in the Netherlands. This implies a need for an evidence-based (international) guideline regarding the treatment of hemorrhoidal disease.

### Author contribution

Robin R. van Tol, M.D.: analysis and interpretation of data, drafting of the article, final approval of article

Marieke P.A. Bruijnen, M.D.: acquisition, analysis and interpretation of data, drafting of the article, final approval of article

Jarno Melenhorst, M.D., Ph.D.: acquisition, analysis and interpretation of data, drafting of the article, final approval of article

Sander M.J. van Kuijk, Ph.D.: analysis and interpretation of data, drafting of the article, final approval of article

Laurents P. Stassen, M.D., Ph.D., Prof.: conception and design, interpretation of data, revision and final approval of article

Stéphanie O. Breukink M.D., Ph.D.: conception and design, interpretation of data, revision and final approval of article

## Appendix 1: Questionnaire

### PERSONAL DATA

- You are a:
  - Consultant
  - Fellow
  - Resident
- Experience in surgery:
  - 1-5 years
  - 5-10 years
  - 10-20 years
  - > 20 years
- Gender: male/ female
- Do you work fulltime or part time?

### DATA HOSPITAL

- Do you work in an Academic/ non-academic/ private hospital?
- When a patient with hemorrhoids visits the outpatient clinic, he is seen by:
  - Consultant
  - Fellow
  - Resident
  - Nurse practitioner
  - Other
- Department of treatment:
  - Surgery department
  - Gastroenterology department
  - Dermatology department
  - Otherwise

### INTERNAL HEMORRHOIDS

- Classification internal hemorrhoids:
  - Goligher classification
  - Otherwise
- Treatment of hemorrhoids grade I, II and III?
  - Rubber Band Ligation: Grade I, II and III
  - Sutured hemorrhoidopexy: Grade I, II and III
  - Stapled hemorrhoidopexy: Grade I, II and III
  - Traditional hemorrhoidectomy: Grade I, II and III
  - Other: Grade I, II and III
- Treatment of grade IV incarcerated hemorrhoids:
  - Conservative
  - Lidocaine ointment
  - ISDN ointment
  - Directly surgical treatment
  - Other

### BARRON LIGATION

- Rubber Band Ligation is performed by the:
  - Consultant
  - Fellow
  - Resident
  - PA of Nurse Practitioner
- Treatment strategy after failed RBL for grade I-IV hemorrhoidal disease
  - One attempt with Barron ligation
  - 2- 3 attempts with Barron ligation
  - 3-4 attempts with Barron ligation
  - 5 or more attempts with Barron ligation
  - Surgical procedure
  - Other

### FIRST TREATMENT

- Which treatment did you perform in grade I-IV?
  - Barron ligation: Grade I, II, III, IV
  - Laser therapy: Grade I, II, III, IV
  - Sutured hemorrhoidopexy: Grade I, II, III, IV
  - Stapled hemorrhoidopexy (PPH/ STARR): Grade I, II, III, IV
  - Doppler-guided hemorrhoidal artery ligation (DGHAL): Grade I, II, III, IV
  - Doppler-guided hemorrhoidal artery ligation with Recto-Anal Repair (DG-HAL+RAR): Grade I, II, III, IV
  - Traditional hemorrhoidectomy Grade I, II, III, IV
  - Otherwise (Referral to another hospital)

### TREATMENT AFTER RECURRENCE

- Which surgical treatment did you perform when RBL failed in grade I, II, III and IV:
  - Laser therapy: Grade I, II, III, IV
  - Sutured hemorrhoidopexy: Grade I, II, III, IV
  - Stapled hemorrhoidopexy (PPH/ STARR): Grade I, II, III, IV
  - Doppler-guided hemorrhoidal artery ligation (DGHAL) : Grade I, II, III, IV
  - Doppler-guided hemorrhoidal artery ligation with Recto-Anal Repair (DG-HAL+RAR): Grade I, II, III, IV
  - Classical hemorrhoidectomy (Milligan-Morgan or Fergusson): Grade I, II, III, IV
  - Otherwise (Referral to another hospital)
- Which treatment did you perform when surgical treatment failed in grade I, II, III and IV?
  - Barron ligation: Grade I, II, III, IV
  - Laser therapy: Grade I, II, III, IV
  - Sutured hemorrhoidopexy: Grade I, II, III, IV
  - Stapled hemorrhoidopexy (PPH/ STARR): Grade I, II, III, IV
  - Doppler-guided hemorrhoidal artery ligation (DGHAL): Grade I, II, III, IV
  - Doppler-guided hemorrhoidal artery ligation with Recto-Anal Repair (DG-HAL+RAR): Grade I, II, III, IV
  - Classical hemorrhoidectomy (Milligan-Morgan or Fergusson): Grade I, II, III, IV
  - Reference to another hospital
  - Otherwise
- When did you perform the traditional hemorrhoidectomy?
  - Regularly performed for all grades of hemorrhoids
  - Only performed at grade III and IV hemorrhoids
  - Only performed at incarcerated hemorrhoids
  - Only performed when other therapies fail
  - Never performed

### COMPLICATIONS

#### Mild complications

- The reported minor complications after use of different surgical treatment options for hemorrhoidal disease:
  - Rubber band ligation
    - Perianal hematoma
    - Delayed wound healing
    - Mild bleeding
    - Mild pain
    - Urinary retention
    - Not applicable
  - Laser therapy
    - Perianal hematoma
    - Delayed wound healing
    - Mild bleeding
    - Mild pain
    - Urinary retention
    - Not applicable
  - Sutured hemorrhoidopexy
    - Perianal hematoma
    - Delayed wound healing
    - Mild bleeding
    - Mild pain
    - Urinary retention
    - Not applicable
  - Doppler-guided hemorrhoidal artery ligation (DG-HAL)
    - Perianal hematoma
    - Delayed wound healing
    - Mild bleeding
    - Mild pain
    - Urinary retention
    - Not applicable
  - Doppler-guided hemorrhoidal artery ligation with Recto-Anal Repair (DG-HAL+RAR)
    - Perianal hematoma
    - Delayed wound healing
    - Mild bleeding
    - Mild pain
    - Urinary retention
    - Not applicable
  - Stapled hemorrhoidopexy (PPH/ STARR)
    - Perianal hematoma
    - Delayed wound healing
    - Mild bleeding
    - Mild pain
    - Urinary retention
    - Not applicable
  - Traditional hemorrhoidectomy
    - Perianal hematoma
    - Delayed wound healing
    - Mild bleeding
    - Mild pain
    - Urinary retention
    - Not applicable
- Most reported mild complication was:
  - Perianal hematoma
  - Delayed wound healing
  - Mild bleeding
  - Mild pain
  - Urinary retention
  - Otherwise
- How often did you see mild complications?
  - 1-5%
  - 5-10%
  - 10-20%
  - >20%

#### Major complications

- The reported major complications after use of different surgical treatment options for hemorrhoidal disease:
  - Rubber band ligation
    - Sepsis
    - Fasciitis necroticans
    - Severe bleeding
    - Admission IC/MC
    - Creating a stoma
    - Severe pain
    - Incontinence
    - Not applicable
  - Laser therapy
    - Sepsis
    - Fasciitis necroticans
    - Severe bleeding
    - Admission IC/MC
    - Creating a stoma
    - Severe pain
    - Incontinence
    - Not applicable
  - Sutured hemorrhoidopexy
    - Sepsis
    - Fasciitis necroticans
    - Severe bleeding
    - Admission IC/MC
    - Creating a stoma
    - Severe pain
    - Incontinence
    - Not applicable
  - Doppler-guided hemorrhoidal artery ligation (DG-HAL)
    - Sepsis
    - Fasciitis necroticans
    - Severe bleeding
    - Admission IC/MC
    - Creating a stoma
    - Incontinence
    - Not applicable
  - Doppler-guided hemorrhoidal artery ligation with Recto-Anal Repair (DG-HAL+RAR)
    - Sepsis
    - Fasciitis necroticans
    - Severe bleeding
    - Admission IC/MC
    - Creating a stoma
    - Severe pain
    - Incontinence
    - Not applicable
  - Stapled hemorrhoidopexy (PPH/ STARR)
    - Sepsis
    - Fasciitis necroticans
    - Severe bleeding
    - Admission IC/MC
    - Creating a stoma
    - Severe pain
    - Incontinence
    - Not applicable
  - Traditional hemorrhoidectomy
    - Sepsis
    - Fasciitis necroticans
    - Severe bleeding
    - Admission IC/MC
    - Creating a stoma
    - Severe pain
    - Incontinence
    - Not applicable
- How often did you see a major complication?
  - 1-5%
  - 5-10%
  - 10-20%
  - >20%

### FOLLOW UP

- What do you use as primary outcome?
  - Absence of complaints
  - Satisfaction of patient
  - Patient does not return to outpatient clinic
  - No further treatment is necessary
  - No abnormalities visible at control proctology
- First visit outpatient clinic after (surgical) treatment: (Fill in the number of weeks)
- Second visit outpatient clinic after (surgical) treatment: (Fill in the number of weeks)
